# Autoimmune Hepatitis: Shifts in Gut Microbiota and Metabolic Pathways among Egyptian Patients

**DOI:** 10.3390/microorganisms8071011

**Published:** 2020-07-06

**Authors:** Nahla M. Elsherbiny, Mohammed Rammadan, Elham A. Hassan, Mohamed E. Ali, Abeer S. Abd El-Rehim, Wael A. Abbas, Mohamed A. A. Abozaid, Ebtisam Hassanin, Helal F. Hetta

**Affiliations:** 1 Medical Microbiology and Immunology Department, Faculty of Medicine, Assiut University, Assiut 71515, Egypt; 2 Microbiology and Immunology Department, Faculty of Pharmacy, Al-Azhar University, Assiut 71524, Egypt; mohamedhussien@azhar.edu.eg (M.R.); elkady4work@azhar.edu.eg (M.E.A.); 3 Gastroenterology and Tropical Medicine Department, Faculty of Medicine, Assiut University, Assiut 71515, Egypt; mam_elham75@yahoo.com (E.A.H.); sharafabeer@yahoo.com (A.S.A.E.-R.); 4 Internal Medicine Department, Gastroenterology and Hepatology Unit, Faculty of Medicine, Assiut University, Assiut 71515, Egypt; Drwaelabbas@yahoo.com (W.A.A.); mabozaid1980@aun.edu.eg (M.A.A.A.); 5 Clinical Pathology, Ministry of Health, Assiut 71511, Egypt; roofy2006@yahoo.com; 6 Internal Medicine Department, College of Medicine, University of Cincinnati, Cincinnati, OH 45267-0595, USA

**Keywords:** bioinformatics, microbiome, autoimmune hepatitis

## Abstract

Autoimmune hepatitis (AIH) is a chronic inflammatory disorder with complex immunopathogenesis. Dysbiosis has been linked to many autoimmune diseases, but its detailed role in autoimmune hepatitis (AIH) still needs rigorous evaluation, especially in Egypt. We aimed to identify the shift in the gut microbiota profile and resultant metabolic pathways in AIH Egyptian patients compared to healthy individuals. Stool samples were collected from 15 AIH-naive patients and from 10 healthy individuals. The V3-V4 hyper-variable regions in16S rRNA gene was amplified and sequenced using Illumina MiSeq platform. Significantly lower bacterial diversity in AIH patients was found compared to the controls. A phylum-level analysis showed the overrepresentation of Firmicutes, Bacteroides, and Proteobacteria. At the genus level, AIH-associated enrichment of *Faecalibacterium*, *Blautia*, *Streptococcus*, *Haemophilus, Bacteroides*, *Veillonella*, *Eubacterium*, *Lachnospiraceae* and *Butyricicoccus* was reported in contrast to *Prevotella*, *Parabacteroides* and *Dilaster*, which were significantly retracted in such patients. Overall, the predicted metabolic pathways associated with dysbiosis in AIH patients could orchestrate the potential pathogenic roles of gut microbiota in autoimmune disease, though not in a disease-specific manner, calling for future large-scale studies.

## 1. Introduction

Autoimmune hepatitis (AIH) is a chronic immune-mediated inflammatory liver disease of uncertain cause [1]. It is characterized histologically by interface hepatitis and the presence of plasma cells, biochemically by the presence of elevated serum transaminase levels, and serologically by increased levels of immunoglobulin G (IgG) with the presence of elevated autoantibodies [2].

AIH can affect any age groups, with type 1 AIH being more common in adults. It is more common in females with a ratio of 3.5 to one and in association with other autoimmune diseases [3]. The clinical severity and outcomes of AIH seem to vary between ethnic populations due to differences in genetic, dietary, and environmental conditions. Progressive hepatic fibrosis occurs in 25% of patients [4].

The immunopathogenesis of AIH is complex and remains unsolved. The principal target antigen in most adults with autoimmune hepatitis is unknown, being an unrecognized self-antigen or a foreign antigen that resembles a self-antigen. It may trigger the disease or increase susceptibility to it by skewing components of the innate and adaptive immune responses toward a proinflammatory and autoreactive profile [1].

The human intestinal mucosal sites are influenced by the surrounding environment and are the sites where millions of microbial residents have emerged as a unique organ that constantly shapes host immunity and metabolism. These commensal bacteria and their metabolic byproducts constitute a reservoir of foreign antigens that can interact with mucosal immune cells and influence systemic immune response and are responsible for the wellbeing of the individual [5]. Recent scientific advances supported additionally by “omics analyses” have been crucial for the generation of a large amount of data relative to the composition of the microbiota [6].

As the liver is physiologically exposed to gut-derived microbial components and metabolites because 70% of its blood supply is derived from the portal vein, intestinal dysbiosis has been associated with many liver diseases, including autoimmune liver disease [7] in experimental animals [8] and in humans [9].

Although much is known regarding the role of gut microbiota in many systemic immune-mediated diseases, their role in the occurrence and behavior of autoimmune hepatitis warrants rigorous evaluation [10]

To the best of our knowledge, the available data associated with intestinal dysbiosis and AIH remain limited in our region. Thus, we aimed to identify the shift in the intestinal microbiota profile and the resultant alteration in the metabolic pathways in Egyptian patients with AIH compared to healthy individuals.

## 2. Materials and Methods

### 2.1. Ethics Statement

This study was approved by the Scientific Research Ethical Committee at the Faculty of Medicine, Assiut University (Institutional Review Board number 17300317/2015), and was conducted according to the principles of the Declaration of Helsinki. Full descriptive and informed consent was taken from each individual prior to sample collection.

### 2.2. Study Design and Participants

Participants in the study included 15 patients with newly diagnosed AIH based on clinical, serological and histological characteristics who were admitted to the Tropical Medicine and Gastroenterology Departments, Assiut University Hospitals, Egypt, from January 2017 to February 2018. The age of the patients ranged from 18 to 36 years. The diagnosis of AIH relied upon the 1999 revised International Autoimmune Hepatitis Group (IAIHG) score ≥ 10 [11] and/or 2008 (IAIHG) simplified AIH score ≥ 7 “definite AIH” [12].

For all patients, a thorough medical history and physical examination were taken for data collection, e.g., age, sex, co-morbidities like diabetes. Laboratory investigations including liver and kidney function tests, complete blood count, and hepatitis B/C serology were done. Exclusion criteria included the use of antibiotics, proton pump inhibitors, steroids or immunosuppressive therapy within the last month prior to inclusion, and any association with other infectious or autoimmune disease.

Ten healthy adults of relevant age, gender and body mass index (BMI) were enrolled as controls. The control group had previously normal laboratory parameters and did not take any of the previously described drugs within the last month before sample collection.

### 2.3. Sample Collection and DNA Extraction

Stool samples were collected from all participants in sterile plastic containers and were transported on ice to the Medical Research Laboratory at Faculty of Medicine, Assiut University, Egypt. Immediate DNA extraction was performed using PureLink™ Microbiome DNA Purification Kit, cat. No. A2979 (Thermofisher Scientific, Waltham, MA, USA) according to the manufacturer’s instructions. Microscopic examination of the stool samples was done to exclude the presence of any parasitic infection.

### 2.4. Polymerase Chain Reaction (PCR) Amplification and Sequencing of V3-V4 in16S rRNA Gene

The immediate amplification of V3-V4 hyper-variable regions in16S rRNA gene by PCR was done. The used primers, which included the Illumina adaptor (underlined) and the PCR cycling conditions, were used as previously reported [13].

Forward Primer:

5′ TCGTCGGCAGCGTCAGATGTGTATAAGAGACAGCCTACGGGNGGCWGCAG 3′ Reverse Primer:

5′GTCTCGTGGGCTCGGAGATGTGTATAAGAGACAGGACTACHVGGGTATCTAATCC′.

The size and quality of PCR amplicons were validated using 1% agarose gel. Amplicons were purified by the Agencourt XP Ampure Beads (BeckamCoulter, Brea, CA, USA). Finally, PCR amplicons of fecal samples and negative controls were sequenced using the Illumina MiSeq platform (Illumina, San Diego, CA, USA) at IGA Technology Services (Udine, Italy).

### 2.5. Data Analysis

All raw sequences generated by Illumina MiSeq were processed through Quantitative Insights into a Microbial Ecology (QIIME) pipeline [14]. In brief, forward and reverse raw sequences were merged, then their quality was checked for the removal of low-quality sequences (Phred score value ≤30 and ≤460 bp), ambiguous reads and potential chimeric sequences. High-quality reads were used for the generation of Operational Taxonomic Units (OTUs) using UCLUST at 97% similarity [15]. The pick_closed_reference_otus script in QIIME was used for the taxonomy assignment of OTUs against the SILVA SSU Ref NR dataset v.132.

Moreover, for the prediction of functional potential of metagenomes, OTU picking was also performed against the Greengenes database (V 13.8) [16] at 97% identity. The resulting biom file was inputted to Phylogenetic Investigation of Communities by Reconstruction of Unobserved States (PICRUSt) by mapping the Kyoto Encyclopedia of Genes and Genome (KEGG) Orthology (KO) Database at level 2 and level 3. The overall taxonomic diversity across the gut microbiomes was estimated using different alpha diversity metrics, based on species richness, which estimates the number of OTUs and Shannon diversity index. Beta diversity analysis was carried out by beta_diversity_through_plots.py to estimate both the unweighted and weighted UniFrac distance matrix. The core microbiome of our dataset represents taxa that are present in 80% of all samples, while the core taxa for each study group represent taxa that were detected in 100% of samples of given group, defined using compute_core_microbiome.py. To define the correlations of bacterial community members to participants’ clinical metadata, the Spearman correlation coefficient was measured for genera whose mean relative abundance ≥ 0.29% using the R package.

### 2.6. Statistical Analysis

For identifying either significantly distinct taxa or metabolic pathways between diseased and healthy subjects, LEfSe (ver. 1.0) software was used (linear discriminative analysis (LDA) scores ≥2 and ≥3 for phylum and genera, respectively) [17]. A permutational multivariate analysis of variance (PERMANOVA) was used to assess the statistical significance of the disease-based clustering of fecal microbiota on principal coordinate analysis (PCoA) plots. The QIIME script group_significance.py was conducted to define the significant differences between study groups by a nonparametric Wilcoxon rank sum test. For multiple comparisons, p-values were adjusted using the false discovery rate (FDR) method in R [18]. The microbiome analyst interface was used for the calculation and plotting of alpha diversity analysis and correlation analysis [19].

### 2.7. Availability of Data

The raw 16SrRNA reads of this study were deposited in the Sequence Read Archive under accession number PRJNA551761 (https://www.ncbi.nlm.nih.gov/bioproject/551761).

## 3. Results

### 3.1. Characteristics of the Study Cohort

Fifteen patients with newly diagnosed AIH (three males and 12 females with a mean age of 27 ± 7.5 years) and 10 healthy controls (three males and seven females with a mean age of 29.3 ± 8 years) were enrolled in the study. The demographic, clinical and laboratory characteristics of the patients are shown in Table 1.

### 3.2. Sequencing of 16S rRNA Gene

Illumina MiSeq platform generated 3,465,502 reads (average count per sample = 138,620; maximum count per sample = 500,834 and minimum count per sample = 66,601), then they were quality checked and filtered and, finally, 2,841,712 reads were used for downstream analysis, including taxonomy assignment, profiling and biodiversity analyses.

### 3.3. Distinct Reduction in Microbial Diversity of AIH Microbiomes

**Figure 1:**
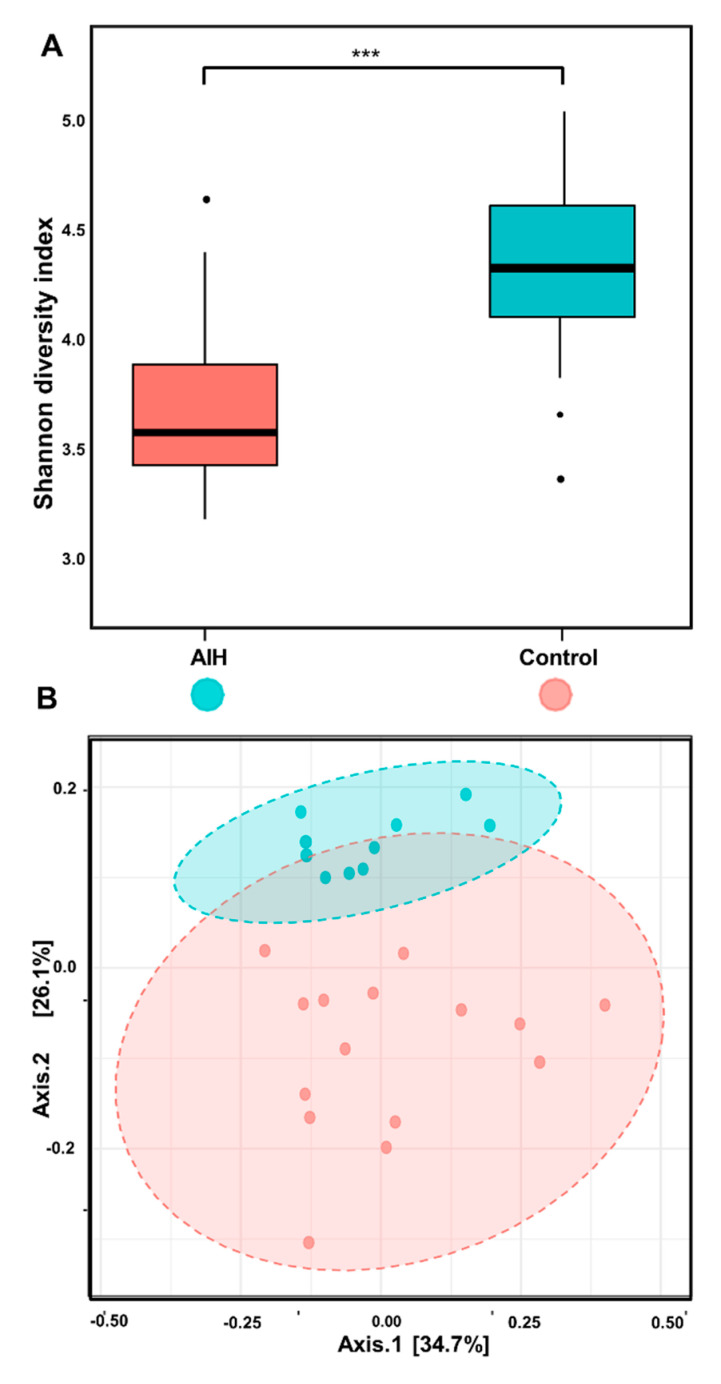
Bacterial diversity analysis of gut microbiota. (**A**) Alpha diversity index (Shannon diversity index) was represented as box plots of (a) for each study group. The median was defined as the line inside each box while interquartile range (IQR) between the25th and 75th percentile was delimited by the outer box. The *x*-axis shows study groups and the *y*-axis represents the Shannon index. A nonparametric Wilcoxon rank sum test was estimated to highlight the statistical significance of pairwise comparisons which was symbolized as either (ns < 1), *** *p* < 0.001. (**B**) Principal coordinate analysis (PCoA) based on weighted UniFrac distance matrix of gut microbial community structures for patients and controls. The first and second coordinates were visualized by the *x*- and *y*-axes, respectively. Community variations were explained by the percentages in parentheses on each axis 34.7% and 26.1%, respectively. Ellipses indicate significant clustering (*p*-value < 0.001, permutational multivariate analysis of variance (PERMANOVA)) according to age. Green and red circles represent healthy controls and patients with autoimmune hepatitis, respectively.

The alpha biodiversity of gut bacterial communities was evaluated using both indices for richness and evenness. Interestingly, the microbiota of AIH were characterized by significantly lower bacterial diversity than the controls in terms of the observed species and Shannon diversity index (Kruskal–Wallis, *p* = 2.29 × 10^−4^) (Figure 1A). Likewise, the structure of the gut microbiota in terms of beta diversity also significantly drove the distinctive disease-dependent clustering of fecal microbiota, especially *Prevotella* and *Veillonella* (Adonis: r2 = 0.042; *p* ≤ 0.001) (Figure 1B).

### 3.4. Remarkable Taxonomic Profile of the Gut Microbiota and Disease-Specific Microbiota Signatures

**Figure 2:**
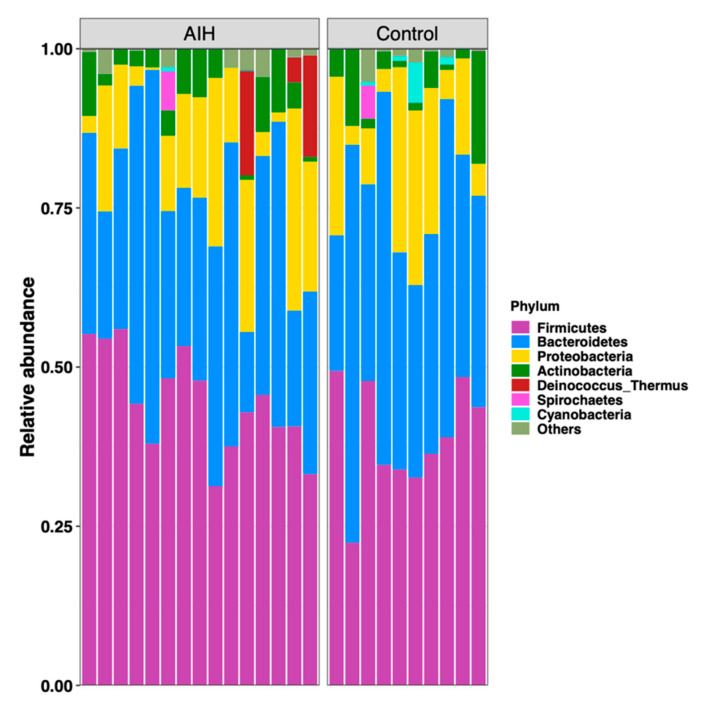
Phylum level analysis of gut microbiota among the studied groups.

Taxonomy profile of gut microbiomes in both study groups exhibited notable patterns at different taxonomic levels, especially at genus and species levels. In total, phylum (23), class (57), order (124) family (246), genus (621) and OTU (5278) were found in all dataset. Phylum level analysis showed an overrepresentation of Firmicutes, Bacteroides, and Proteobacteria (representing about 98.31% of all reads) (Figure 2). Regardless of the health state, the Firmicutes/Bacteroides ratio (F/B) was significantly variable between samples. On the other hand, F/B ratios of those with AIH were significantly linked to total bilirubin (F/B = 1.72, *p* = 0.001).

**Figure 3:**
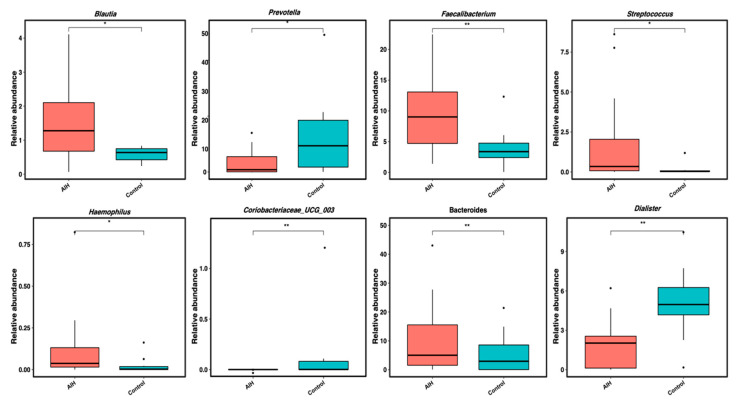
Genera that significantly differed between the studied groups. Box plots display the dominant genera that were detected with significant differences in abundance between studied age groups. Asterisks denote significant differences (ns, *p* > 1; * *p* < 0.05; ** *p* < 0.01).

At genus level, the overall representation of the most abundant genera (detected with a relative abundance ≥ 0.11 of reads) showed the disease-associated enrichment of certain genera (Figure 3). For instance, the genera *Faecalibacterium*, *Blautia*, *Streptococcus* (which belong to the Firmicutes phylum), *Bacteroides* (belonging to the Bacteroidetes phylum) and *Haemophilus* (belonging to the Proteobacteria phylum) were markedly enriched in AIH patients in contrast to *Prevotella*, *Parabacteroides* (Bacteroidetes phylum) and *Dilaster* (Firmicutes phylum) that were significantly retracted in such patients (Figure 3). In addition, the linear discriminative analysis (LDA) based on LEfSe highlighted the significant differentiating enrichment of *Veillonella*, *Eubacterium*, *Lachnospiraceae* and *Butyricicoccus* (all are members of the Firmicutes phylum) in the AIH group.

The surveying taxa or OTUs that were shared between all samples or between each study group identified distinct bacterial signatures for each health state. Noticeably, there were 23 genera found as core microbiomes for all datasets that included the OTUs affiliated with the major phyla in addition to low-abundance taxa such as *Lachnoclostridium* (Table 2).

The bar charts denote the relative representation of most abundant phyla in the fecal microbiome of patients with AIH and in healthy controls.

### 3.5. Functional Profile of Gut Microbiota

PICRUSt was used to infer the collective functional potential of gut metagenomes. Overall, 23 metabolic pathways associated with cellular process, amino acid metabolism and lipid metabolisms were differentially overrepresented in those with AIH in contrast to healthy controls (LDA score >2.0, *p* < 0.05) (Supplemental Table S1). Notably, the overrepresentation of butyrate, tryptophan, branched-chain fatty acids, pantothenate and coenzyme A metabolism in microbial communities was associated with AIH. Furthermore, metabolic modules associated to proline and arginine were significantly underrepresented.

### 3.6. Microbe-Microbe Interactions Strongly Associated with AIH Pathogenesis

Variable intercommunity interactions were exhibited across different taxonomic levels. A notable positive coexistence was found between different taxa that formed distinct blocks (Supplemental Figure S1). For instance, at genus level, *Coprococcus*, *Eubacterium*, *Ruminococcus* and *Roseburia* represented the strongest correlation in the entire dataset. Moreover, this cluster of genera was relatively correlated to the *Barnesiella* and other members of *Prevotella*. Moreover, phylum level analysis markedly determined the intercommunity competition, especially between the most abundant phyla.

## 4. Discussion

The evident linkage between dysbiosis and human autoimmune hepatitis has been reported [8,9]. The major uncertainty has been whether the dysbiosis has been a cause or an effect of the disease. This disorder has been associated with increased permeability of the gastrointestinal mucosal barrier, and the translocation of gut-derived microbial products into systemic circulation [9].

In the current study, AIH occurred predominately in females, as was previously reported [20]. An outstanding reduction in bacterial diversity was significantly observed in AIH microbiomes regarding evenness and richness (Kruskal–Wallis, *p* = 2.29 × 10^−4^). Similarly, the gut microbiota of steroid treatment-naive AIH were characterized by lower alpha diversity (Shannon and observed operational taxonomic units, both *p* < 0.01) and a distinct overall microbial composition compared with healthy controls (*p* = 0.002) [21]. Interestingly, an obvious lowering in microbial diversity was observed in many autoimmune diseases such as type I diabetes [22], rheumatoid arthritis [23] and inflammatory bowel disease [24]. Additionally, inter-individual variations in our dataset may be principally attributed to enterotypes, body mass index (BMI) level, and external factors such as lifestyle, exercise frequency, ethnicity, and dietary and cultural habits [25].

Moreover, the disease-dependent clustering of microbiomes was highlighted by a PCoA plot that was mainly driven by *Prevotella* and *Veillonella* (Adonis: r2 = 0.042; *p* ≤ 0.001). In agreement, *Veillonella* was reported to be increased in patients with type I dependent diabetes mellitus [22] and and Crohn’s disease [26]. A significant correlation between the relative abundance of *Veillonella* and inflammatory markers (e.g., h-CRP) was proven, pointing to its role in psoriasis (an autoimmune disease of the skin) [27]. In addition to the association evidence with several inflammatory conditions, murine models have demonstrated a central role of *Veillonella* in the promotion of inflammation or the perturbation of immune homeostasis within and beyond the intestine [28]. Taken together, it is plausible that *Veillonella* spp. contributed to the activation of hepatic inflammation, although the effect may not be disease specific [21]. On the contrary, it was increased in patients with systemic lupus erythematosus (SLE) [29].

The overrepresentation of Firmicutes, Bacteroidetes, and Proteobacteria in the entire dataset (representing about 98.31% of all reads) was also previously reported to be the most prominent amongst patients with psoriasis and controls [27]. Although the Firmicutes/Bacteroidetes ratio (F/B) was significantly variable between samples in the present study, regardless of the health state, F/B ratios of patients with AIH were significantly linked to total bilirubin, as many bile acid metabolizing species belong to the Firmicutes phylum [30]. With the exception of the study on systemic lupus erythematosus [5], B/F ratio was not increased in other autoimmune diseases, in agreement with this study.

In accordance with the study on multiple sclerosis, *Veillonella*, *Eubacterium*, *Lachnospiraceae* and *Butyricicoccus* were significantly enriched in AIH patients [31].

Furthermore, we reported an increase in the proportions of *Haemophilus* and *Blautia* genera and a reduction in *Parabacteroides*. In contrast to this study, a previous study demonstrated a significant decrease in *Bifidobacterium* and *Lactobacillus* in AIH patients compared with healthy subjects, while no changes were observed in *Escherichia coli* and *Enterococcus* [9].

The degree of dysbiosis differs according to the course of the disease, whether in remission or relapse. In accordance with the results of this study, certain genera such as *Blautia*, *Dorea* and *Haemophilus* were enriched in relapsing and remitting multiple sclerosis (RRMS) patients [32].

There is a very complex host–microbiota interaction that makes the understanding of the effect of dysbiosis in the occurrence of autoimmunity very difficult. A species may need help from other microbes to produce an effect, thereby not being the perpetrator of the effect, or different strains may differ in their ability to affect the development of an autoimmune disease, as in autoimmune diabetes [33]. Moreover, the host genetics may be a decisive factor in the ability of an organism to produce a certain effect [34]. In addition, the microbiota can cause opposing effects in different situations [33].

An important finding is the increased proportions of genus *Faecalibacterium* and butyrate-producing bacteria such as *Butyricicoccus, Ruminococcaceae* (also referred as clostridial cluster IV) and *Lachnospiraceae* (also referred to as clostridial cluster XIVa) [35], which were defined as members of the core microbiome in this study, in addition to the overrepresentation of metabolic modules associated to butyrate metabolism in AIH subjects. Although a relatively large group of *Clostridia* can induce Tregs, it is not known whether some members of the group do it better than others or if the whole microbial consortium is needed to induce the response [36]. *Faecalibacterium* spp. are well known for their production of anti-inflammatory molecules as well as butyrate, which could prevent systemic immune responses [37]. The role of butyrate in gastrointestinal permeability and integrity, in addition to the development of peripheral tolerance through production of Treg cells, is well known [38]. Surprisingly, butyrate and other SCFs have been recently reported to have a dual role in the pathogenesis of autoimmune disease models, ameliorating the disease severity of lymphocyte-mediated systemic autoimmune inflammatory conditions mediated by lymphocytes such as collagen-induced arthritis and experimental autoimmune encephalitis through a reduction in Th1 cells and an increase in regulatory T cells and, on the other hand, exaggerating the effector phase of inflammation in rheumatoid arthritis. It was reported that butyrate, unexpectedly, enhanced innate cell-mediated inflammation, augmenting the development antibody-induced arthritis in mice and increasing the disease severity clinically and histologically [39]. It was also found that orally administered SCFAs increase Th17 in mice [38,39]. This is completely different from the previous study demonstrating that butyrate treatment reduced the production of pro-inflammatory cytokines IL-6 and IL-1β and increased IL-10 production from the LPS-stimulated murine macrophage cell line [40].

Although butyrate is well known to repair and enhance the barrier function of intestinal epithelial cells by many mechanisms [41], in vitro models showed that the effect of butyrate on the intestinal barrier function may be concentration dependent [42]. That is to say, at low concentrations (≤2 mM), it promotes intestinal barrier function [43], while at high concentrations (5 or 8 mM), it may disrupt such barrier function by inducing the apoptosis of the epithelial cells [42]. In addition, the modulation of the intestinal epithelial cell-mediated migration of neutrophils to inflammatory sites is also concentration dependent [44]. Thus, the enrichment of many butyrate-producing members among the microbiome of the AIH patients in this study probably produce high concentrations of butyrate, disrupting the intestinal barrier functions and epithelial cell-mediated migration of neutrophils, contributing to the pathogenesis of the disease.

The pathogenesis of AIH is not fully clear, though genetic susceptibility is considered to be the main factor [45]. Acute flare-ups of AIH are driven by innate immune responses such as natural killer (NK) cells and innate lymphoid cells, while chronic active AIH is characterized by an effector CD4 and CD8T cell immune response [46]. Under normal conditions, the immune response to autoantigens is not evoked because of immune tolerance. Once a liver autoantigen is recognized, naive T cells are activated and differentiated into Th1, Th2, or Th17 cells, depending on the immunological microenvironment and the nature of the antigen, and the immune reaction is initiated. Th17 cells are considered to be a major cause of autoimmune liver disease. These cells can also suppress Tregs [47]. As many butyrate-producing bacteria were enriched among the microbiota of the AIH patients in this study, the probable high concentration of resultant butyrate may increase TH17, as was previously reported in mice [38,39].

It is paradoxical to note that the family *Coriobacteriaceae*, which was linked to the metabolism of bile acids [48], was retracted in abundance in AIH compared to healthy controls. Surprisingly, the overrepresentation of *Eubacterium* and its positive correlation to bilirubin levels in AIH could be considered as a compensatory response to the depletion of *Coriobacteriaceae* in AIH subjects [49]. Likewise, *Bacteroides, Clostridium, Lactobacillus, Bifidobacterium* and *Eubacterium* were found to be enriched among the AIH patients in this study. They affect bile acid metabolism through different pathways, raising the possibility of a compensatory mechanism played by the gut microbiota to deal with the increased BAs in AIH patients, supporting the altered liver–microbiota–bile acid crosstalk [50].

Regarding the key metabolic changes among patients with AIH in this study, metabolic pathways associated with cellular process, amino acid metabolism and lipid metabolisms were differentially overrepresented in those with AIH in contrast to healthy controls. Valine, Leucine and Isoleucine are branched-chain amino acids (BCAA) that regulate many key signaling pathways, participate in intestinal barrier function and upregulate innate and adaptive immune responses [51]. The metabolism of such BCAAs, in addition to the pantothenate and coenzyme A biosynthesis pathway, were significantly increased in the AIH group in this study. In agreement, a previous study, reported an increase in the cirrhosis-related proteins associated with two metabolic pathways, which were map00290 (valine, leucine and isoleucine biosynthesis) and map00770 (pantothenate and coenzyme A biosynthesis) [52].

In general, the study of Chang et al. [53] reported similar changes in experimentally induced liver fibrosis in rats, where changes occurred during liver fibrosis initiation and progression. As a result, the accurate modulation of amino acid metabolism can play a relevant role in the prevention or treatment of autoimmune diseases [54]. Moreover, tryptophan metabolism was significantly noted in the AIH group in the current study, which is in accordance with the recent study ensuring that impaired tryptophan metabolism by the gut microbiota was associated with a susceptibility to colonic inflammation [55]. Regarding arginine (Arg) and proline metabolism, both were significantly higher among controls in this study. This is in agreement with known functions of Arg metabolism as a critical regulator of immune responses, in addition to modulating the production of antimicrobial effectors and directing the activity of T cells [56].

Furthermore, an improper activation of the arginine pathway to produce citrulline may potentiate an immune reaction against any citrullinated proteins and thus participate in many disorders, including autoimmune diseases [54].

In the current study, one of the important carbohydrate metabolic pathways significantly increased in the AIH group is pyruvate metabolism. Pyruvate can be either be converted to acetyl-CoA (which fuels the tricarboxylic acid cycle and leads to lipogenesis) or oxaloacetate (which leads to gluconeogenesis) by two different enzymes. It was recently reported that the targeting of molecules in these pathways was found to treat nonalcoholic steatohepatitis (NASH) [57].

Improving our knowledge of the effect of gut microbial metabolism on liver health could aid in the development of novel microbiota-targeted interventions

Regardless of having a relatively small sample size, the results are in parallel with those of other larger studies, shedding light on some altered metabolic pathways. This being the only study in Egypt to discuss this issue, which is also not commonly studied worldwide, adds to its value.

## 5. Conclusions

This pilot study shows a clear evidence of the association between AIH discriminative microbiota taxa and metabolic pathways. Although the predicted metabolic capabilities of gut microbiota were not disease specific, they do refer to the potential roles in the pathogenesis of the autoimmune process. This demands further large scale studies in addition to the follow up of the AIH patients to evaluate the progress of disease severity.

## Figures and Tables

**Table 1:** Demographic, clinical and laboratory characteristics of the participants.

Parameters	AIH (*n* = 15)	Control (*n* = 10)
Age	27 ± 7.5 (18–36)	29.3 ± 8 (19–40)
Sex male/Female	3/12 (20/80%)	3/7 (30/70%)
Jaundice	7 (46.7%)	NA
Fever	6 (40%)	NA
Ascites	3 (20%)	NA
Severity of the disease Chronic hepatitis Liver cirrhosis	11 (73.3%) 4 (26.7%)	NA
Serum bilirubin (umol/L)	19.2 (5.8–86.9)	NA
Albumin (g/dL)	3.6 ± 0.5	NA
AST (U/L)	39.1 (12.3–136)	NA
ALT (U/L)	43 (11.3–315)	NA
ALP (U/L)	134 (63–301)	NA
WBCs (×10^9^/L)	11.1 ± 3.7	NA
Hb (gm/dL)	10.2 ± 1.5	NA
PLT (×10^9^/L)	265 ± 68.2	NA
Prothrombin time (s)	14.5 ± 3.7	NA
INR	1.3 ± 0.3	NA
Serum creatinine (µmol/L)	50.1 ± 16.8	NA

Nominal data are expressed in the form of frequency (%), while continuous data are expressed in mean ± SD or median and range, as appropriate.

**Table 2:** The core and the shared genera in the entire dataset.

Core Genera for Dataset	Control	AIH
Prevotella_9	Lachnospiraceae_UCG_004	Clostridium_innocuum_group
Bacteroides	Pseudobutyrivibrio	Erysipelatoclostridium
Ruminococcus_gnavus_group	Shimwellia	Lactobacillus
Faecalibacterium	Haemophilus	Uncultured affiliated to Ruminococcus1
Escherichia/Shigella	Eubacterium_hallii_group	Coprococcus_2
Lachnoclostridium	Ruminococcaceae_UCG_002	Eubacterium_coprostanoligenes_group
Agathobacter	Eubacterium_ruminantium_group	Bifidobacterium
Phascolarctobacterium	Lachnospiraceae_NK4A136_group	Holdemanella
Blautia	Parabacteroides	Rikenellaceae_RC9_gut_group
Enterobacter	Ruminococcaceae_UCG_003	Dorea
Streptococcus	Parasutterella	Prevotellaceae_NK3B31_group
Anaeroplasma	Christensenellaceae_R_7_group	Veillonella
Megasphaera	Coriobacteriaceae_UCG_003	Ruminococcus_1
Megamonas		Butyricicoccus
Roseburia		
Collinsella		
Lachnospira		
Catenibacterium		
Subdoligranulum		
Fusobacterium		
Flavonifractor		
Ruminococcaceae_UCG_013		
Dialister		
Ruminococcus_torques_group		

## Supplementary Materials

Supplemental material are available online at https://www.mdpi.com/2076-2607/8/7/1011/s1.

## Abbreviations

AIH	Autoimmune hepatitis
ALP	Alkaline phosphatase
ALT	Alanine transaminase
AST	Aspartate aminotransferase
Hb	Hemoglobin
INR	International normalized ratio
NA	Not applicable
PLT	Platelet count
WBCs	White blood cells

## References

[1] Czaja A.J. (2015). Transitioning from Idiopathic to Explainable Autoimmune Hepatitis. Digest. Dis. Sci..

[2] Czaja A.J., Donaldson P.T. (2002). Gender Effects and Synergisms with Histocompatibility Leukocyte Antigens in Type 1 Autoimmune Hepatitis. Am. J. Gastroenterol..

[3] Muratori P., Fabbri A., Lalanne C., Lenzi M., Muratori L. (2015). Autoimmune Liver Disease and Concomitant Extrahepatic Autoimmune Disease. Eur. J. Gastroenterol. Hepatol..

[4] Czaja A.J., Carpenter H.A. (2004). Progressive Fibrosis during Corticosteroid Therapy of Autoimmune Hepatitis. Hepatology.

[5] Sánchez B., Hevia A., González S., Margolles A. (2015). Interaction of Intestinal Microorganisms with the Human Host in the Framework of Autoimmune Diseases. Front. Immunol..

[6] Almonacid D.E., Kraal L., Ossandon F.J., Budovskaya Y.V., Cardenas J.P., Bik E.M., Goddard A.D., Richman J., Apte Z.S. (2017). 16S RRNA Gene Sequencing and Healthy Reference Ranges for 28 Clinically Relevant Microbial Taxa from the Human Gut Microbiome. PLoS ONE.

[7] Trivedi P.J., Adams D.H. (2013). Mucosal Immunity in Liver Autoimmunity: A Comprehensive Review. J. Autoimm..

[8] Yuksel M., Wang Y., Tai N., Peng J., Guo J., Beland K., Lapierre P., David C., Alvarez F., Colle I. (2015). A Novel “Humanized Mouse” Model for Autoimmune Hepatitis and the Association of Gut Microbiota with Liver Inflammation. Hepatology.

[9] Lin R., Zhou L., Zhang J., Wang B. (2015). Abnormal Intestinal Permeability and Microbiota in Patients with Autoimmune Hepatitis. Int. J. Clin. Exp. Pathol..

[10] Czaja A.J. (2016). Factoring the Intestinal Microbiome into the Pathogenesis of Autoimmune Hepatitis. World J. Gastroenterol..

[11] Alvarez F., Berg P.A., Bianchi F.B., Bianchi L., Burroughs A.K., Cancado E.L., Chapman R.W., Cooksley W.G.E., Czaja A.J., Desmet V.J. (1999). International Autoimmune Hepatitis Group Report: Review of Criteria for Diagnosis of Autoimmune Hepatitis. J. Hepatol..

[12] Hennes E.M., Zeniya M., Czaja A.J., Parés A., Dalekos G.N., Krawitt E.L., Bittencourt P.L., Porta G., Boberg K.M., Hofer H. (2008). Simplified Criteria for the Diagnosis of Autoimmune Hepatitis. Hepatology.

[13] Ramadan M., Solyman S., Taha M., Hanora A. (2016). Preliminary Characterization of Human Skin Microbiome in Healthy Egyptian Individuals. Cell. Mol. Biol..

[14] Caporaso J.G., Kuczynski J., Stombaugh J., Bittinger K., Bushman F.D., Costello E.K., Fierer N., Pẽa A.G., Goodrich J.K., Gordon J.I. (2010). QIIME Allows Analysis of High-Throughput Community Sequencing Data. Nat. Methods.

[15] Edgar R.C., Bateman A. (2010). Search and Clustering Orders of Magnitude Faster than BLAST. Bioinform. Appl. NOTE.

[16] McDonald D., Price M.N., Goodrich J., Nawrocki E.P., Desantis T.Z., Probst A., Andersen G.L., Knight R., Hugenholtz P. (2012). An Improved Greengenes Taxonomy with Explicit Ranks for Ecological and Evolutionary Analyses of Bacteria and Archaea. ISME J..

[17] Segata N., Waldron L., Ballarini A., Narasimhan V., Jousson O., Huttenhower C. (2012). Metagenomic Microbial Community Profiling Using Unique Clade-Specific Marker Genes. Nat. Methods.

[18] Benjamini Y., Hochberg Y. (1995). Controlling the False Discovery Rate: A Practical and Powerful Approach to Multiple Testing. J. R. Stat. Soc. Ser. B Methodol..

[19] Dhariwal A., Chong J., Habib S., King I.L., Agellon L.B., Xia J. (2017). MicrobiomeAnalyst: A Web-Based Tool for Comprehensive Statistical, Visual and Meta-Analysis of Microbiome Data. Nucleic Acids Res..

[20] Czaja A.J. (2017). Global Disparities and Their Implications in the Occurrence and Outcome of Autoimmune Hepatitis. Digest. Dis. Sci..

[21] Wei Y., Li Y., Yan L., Sun C., Miao Q., Wang Q., Xiao X., Lian M., Li B., Chen Y. (2020). Alterations of Gut Microbiome in Autoimmune Hepatitis. Gut.

[22] Alkanani A.K., Hara N., Gottlieb P.A., Ir D., Robertson C.E., Wagner B.D., Frank D.N., Zipris D. (2015). Alterations in Intestinal Microbiota Correlate with Susceptibility to Type 1 Diabetes. Diabetes.

[23] Chen J., Wright K., Davis J.M., Jeraldo P., Marietta E.V., Murray J., Nelson H., Matteson E.L., Taneja V. (2016). An Expansion of Rare Lineage Intestinal Microbes Characterizes Rheumatoid Arthritis. Genome Med..

[24] Alekseyenko A.V., Perez-Perez G.I., De Souza A., Strober B., Gao Z., Bihan M., Li K., Methé B.A., Blaser M.J. (2013). Community Differentiation of the Cutaneous Microbiota in Psoriasis. Microbiome.

[25] Arumugam M., Raes J., Pelletier E., Le Paslier D., Yamada T., Mende D.R., Fernandes G.R., Tap J., Bruls T., Batto J.M. (2011). Enterotypes of the Human Gut Microbiome. Nature.

[26] Gevers D., Kugathasan S., Denson L.A., Vázquez-Baeza Y., Van Treuren W., Ren B., Schwager E., Knights D., Song S.J., Yassour M. (2014). The Treatment-Naive Microbiome in New-Onset Crohn’s Disease. Cell Host Microbe.

[27] Huang L., Gao R., Yu N., Zhu Y., Ding Y., Qin H. (2019). Dysbiosis of Gut Microbiota Was Closely Associated with Psoriasis. Sci. China Life Sci..

[28] Manfredo Vieira S., Hiltensperger M., Kumar V., Zegarra-Ruiz D., Dehner C., Khan N., Costa F.R.C., Tiniakou E., Greiling T., Ruff W. (2018). Translocation of a Gut Pathobiont Drives Autoimmunity in Mice and Humans. Science.

[29] Mendonça S.M.S., Corrêa J.D., de Souza A.F., Travassos D.V., Calderaro D.C., Rocha N.P., Vieira É.L.M., Teixeira A.L., Ferreira G.A., da Silva T.A. (2019). Immunological Signatures in Saliva of Systemic Lupus Erythematosus Patients: Influence of Periodontal Condition. Clin. Exp. Rheumatol..

[30] Ridlon J.M., Kang D.J., Hylemon P.B. (2006). Bile Salt Biotransformations by Human Intestinal Bacteria. J. Lipid Res..

[31] Cekanaviciute E., Yoo B.B., Runia T.F., Debelius J.W., Singh S., Nelson C.A., Kanner R., Bencosme Y., Lee Y.K., Hauser S.L. (2017). Gut Bacteria from Multiple Sclerosis Patients Modulate Human T Cells and Exacerbate Symptoms in Mouse Models. Proc. Natl. Acad. Sci. USA.

[32] Chen J., Chia N., Kalari K.R., Yao J.Z., Novotna M., Soldan M.M.P., Luckey D.H., Marietta E.V., Jeraldo P.R., Chen X. (2016). Multiple Sclerosis Patients Have a Distinct Gut Microbiota Compared to Healthy Controls. Sci. Rep..

[33] Chervonsky A.V. (2013). Microbiota and Autoimmunity. Cold Spring Harb. Perspect. Biol..

[34] Geuking M.B., Cahenzli J., Lawson M.A.E., Ng D.C.K., Slack E., Hapfelmeier S., McCoy K.D., Macpherson A.J. (2011). Intestinal Bacterial Colonization Induces Mutualistic Regulatory T Cell Responses. Immunity.

[35] Geirnaert A., Calatayud M., Grootaert C., Laukens D., Devriese S., Smagghe G., De Vos M., Boon N., Van De Wiele T. (2017). Butyrate-Producing Bacteria Supplemented in Vitro to Crohn’s Disease Patient Microbiota Increased Butyrate Production and Enhanced Intestinal Epithelial Barrier Integrity. Sci. Rep..

[36] Atarashi K., Tanoue T., Shima T., Imaoka A., Kuwahara T., Momose Y., Cheng G., Yamasaki S., Saito T., Ohba Y. (2011). Induction of Colonic Regulatory T Cells by Indigenous Clostridium Species. Science.

[37] Sheng L., Jena P.K., Hu Y., Liu H.X., Nagar N., Kalanetra K.M., French S.W., French S.W., Mills D.A., Wan Y.J.Y. (2017). Hepatic Inflammation Caused by Dysregulated Bile Acid Synthesis Is Reversible by Butyrate Supplementation. J. Pathol..

[38] Haghikia A., Jörg S., Duscha A., Berg J., Manzel A., Waschbisch A., Hammer A., Lee D.H., May C., Wilck N. (2015). Dietary Fatty Acids Directly Impact Central Nervous System Autoimmunity via the Small Intestine. Immunity.

[39] Mizuno M., Noto D., Kaga N., Chiba A., Miyake S. (2017). The Dual Role of Short Fatty Acid Chains in the Pathogenesis of Autoimmune Disease Models. PLoS ONE.

[40] Wang F., Liu J., Weng T., Shen K., Chen Z., Yu Y., Huang Q., Wang G., Liu Z., Jin S. (2017). The Inflammation Induced by Lipopolysaccharide Can Be Mitigated by Short-Chain Fatty Acid, Butyrate, through Upregulation of IL-10 in Septic Shock. Scand. J. Immunol..

[41] Huang C., Song P., Fan P., Hou C., Thacker P., Ma X. (2015). Dietary Sodium Butyrate Decreases Postweaning Diarrhea by Modulating Intestinal Permeability and Changing the Bacterial Communities in Weaned Piglets. J. Nutr..

[42] Huang X.Z., Li Z.R., Zhu L.B., Huang H.Y., Hou L.L., Lin J. (2014). Inhibition of P38 Mitogen-Activated Protein Kinase Attenuates Butyrate-Induced Intestinal Barrier Impairment in a Caco-2 Cell Monolayer Model. J. Pediatr. Gastroenterol. Nutr..

[43] Peng L., Li Z.-R., Green R.S., Holzman I.R., Lin J. (2009). Butyrate Enhances the Intestinal Barrier by Facilitating Tight Junction Assembly via Activation of AMP-Activated Protein Kinase in Caco-2 Cell Monolayers. J. Nutr..

[44] Vinolo M.A.R., Rodrigues H.G., Hatanaka E., Hebeda C.B., Farsky S.H.P., Curi R. (2009). Short-Chain Fatty Acids Stimulate the Migration of Neutrophils to Inflammatory Sites. Clin. Sci..

[45] Wang M., Zhang H. (2018). The Pathogenesis of Autoimmune Hepatitis. Front. Lab. Med..

[46] WANG Q.Y., JIA J.D. (2011). Advances in the Pathogenesis of Autoimmune Hepatitis. J. Clin. Hepatol..

[47] Yeoman A.D., Heneghan M.A. (2010). Anti TNF-α Therapy Can Be a Novel Treatment Option in Patients with Autoimmune Hepatitis: Authors’ Reply. Aliment. Pharmacol. Ther..

[48] Just S., Mondot S., Ecker J., Wegner K., Rath E., Gau L., Streidl T., Hery-Arnaud G., Schmidt S., Lesker T.R. (2018). The Gut Microbiota Drives the Impact of Bile Acids and Fat Source in Diet on Mouse Metabolism. Microbiome.

[49] Swann J.R., Want E.J., Geier F.M., Spagou K., Wilson I.D., Sidaway J.E., Nicholson J.K., Holmes E. (2011). Systemic Gut Microbial Modulation of Bile Acid Metabolism in Host Tissue Compartments. Proc. Natl. Acad. Sci. USA.

[50] Jiao N., Baker S.S., Chapa-Rodriguez A., Liu W., Nugent C.A., Tsompana M., Mastrandrea L., Buck M.J., Baker R.D., Genco R.J. (2018). Suppressed Hepatic Bile Acid Signalling despite Elevated Production of Primary and Secondary Bile Acids in NAFLD. Gut.

[51] Zhang S., Zeng X., Ren M., Mao X., Qiao S. (2017). Novel Metabolic and Physiological Functions of Branched Chain Amino Acids: A Review. J. Anim. Sci. Biotechnol..

[52] Wei X., Jiang S., Chen Y., Zhao X., Li H., Lin W., Li B., Wang X., Yuan J., Sun Y. (2016). Cirrhosis Related Functionality Characteristic of the Fecal Microbiota as Revealed by a Metaproteomic Approach. BMC Gastroenterol..

[53] Chang H., Meng H.Y., Liu S.M., Wang Y., Yang X.X., Lu F., Wang H.Y. (2017). Identification of Key Metabolic Changes during Liver Fibrosis Progression in Rats Using a Urine and Serum Metabolomics Approach. Sci. Rep..

[54] Mondanelli G., Iacono A., Carvalho A., Orabona C., Volpi C., Pallotta M.T., Matino D., Esposito S., Grohmann U. (2019). Amino Acid Metabolism as Drug Target in Autoimmune Diseases. Autoimm. Rev..

[55] Lamas B., Richard M.L., Leducq V., Pham H.P., Michel M.L., Da Costa G., Bridonneau C., Jegou S., Hoffmann T.W., Natividad J.M. (2016). CARD9 Impacts Colitis by Altering Gut Microbiota Metabolism of Tryptophan into Aryl Hydrocarbon Receptor Ligands. Nat. Med..

[56] Murray P.J. (2016). Amino Acid Auxotrophy as a System of Immunological Control Nodes. Nat. Immunol..

[57] McCommis K.S., Finck B.N. (2019). Treating Hepatic Steatosis and Fibrosis by Modulating Mitochondrial Pyruvate Metabolism. CMGH.

