# Supplementary material for: Autoimmune Hepatitis: Shifts in Gut Microbiota and Metabolic Pathways among Egyptian Patients
Source: Microorganisms. 2020 Jul 6;8(7):1011. doi: 10.3390/microorganisms8071011 (PMC7409351; doi:10.3390/microorganisms8071011)
Supplement: Supplementary file 1 [file microorganisms-08-01011-s001.pdf]

microorganisms

MDPI

Supplemental Table S1. Most abundant metabolic pathways among the studied groups.

	Metabolic pathway		LDA score	Study group	P value
Amino acid metabolism	Lysine degradation		3.227744	AIH	< 0.001
	Tryptophan metabolism		3.256763	AIH	< 0.001
	Valine	Leucine and Isoleucine degradation	3.366401	AIH	< 0.0017
	Alanine	Aspartate and Glutamate metabolism	4.043324	AIH	< 0.001
	Lysine biosynthesis		3.744664	AIH	< 0.001
	Phenylalanine metabolism		3.277223	AIH	< 0.001
	Valine	Leucine and Isoleucine biosynthesis	3.670123	AIH	< 0.001
	Arginine and Proline metabolism		4.074334	Control	< 0.001
	amino acid related enzymes		4.226563	Control	< 0.001
	Cysteine and Methionine		4.012464	Control	< 0.001
	Glycine	Serine and Threonine metabolism	3.753356	Control	< 0.001
Biosynthesis of other secondary metabolites	phenyl propanoid biosynthesis		3.247322	AIH	0.001
	isoquinoline alkaloid biosynthesis		2.762526	Control	< 0.001
Carbohydrate metabolism	ascorbate and aldarate metabolism		3.014636	AIH	< 0.001
	inositol phosphate metabolism		2.736357	AIH	< 0.001
	C5 braChed dibasic acid metabolism		3.526336	AIH	< 0.001
	fructose and mannose metabolism		4.001524	AIH	< 0.001
	galactose metabolism		3.663353	AIH	< 0.001
	butanoate metabolism		4.05263	AIH	< 0.001
	glyoxylate and dicarboxylat metabolism		3.327224	AIH	< 0.001
	pentose and glucuronate interconversions		3.342242	AIH	< 0.001
	pentose phosphate pathway		3.756206	AIH	< 0.001
	propanoate metabolism		3.325043	AIH	< 0.001
	pyruvate metabolism		4.013353	AIH	< 0.001
	starch and sucrose metabolism		4.012332	AIH	< 0.001
	citrate cycle TCA cycle		3.673465	Control	0.004
Cellular processes and signaling	cell motility cytoskeleton proteins		3.622344	AIH	0.001
	cell motility	bacterial motility proteins	3.766252	Control	< 0.001
	cell motility	flagellar assembly	3.562233	Control	0.005
	cell growth and death	cell cycle caulobacter	3.364266	Control	< 0.001
	other transporters		3.450126	AIH	< 0.001
	sporulation		3.723263	AIH	0.001
	cell motility and secretion		3.306704	Control	< 0.001
	membrane and intracellular structural molecules		3.663236	Control	0.001
	pores ion channels		3.665453	Control	0.001
endocrine system	insulin signaling pathway		2.777766	AIH	< 0.001
Energy metabolism	methane metabolism		4.225306	AIH	< 0.001
	carbon fixation pathways in prokaryotes		4.013663	Control	0.01
	photosynthesis		3.67244	Control	< 0.001
	photosynthesis proteins		3.673225	Control	< 0.001
Environme ntal information processing	processing membrane transport secretion system		4.201323	Control	< 0.001
	membrane transport phosphor transferase system		3.56562	AIH	< 0.001

	membrane transport transporters		4.601674	AIH	0.01
	signaling molecules and interaction bacterial toxins		3.066464	AIH	< 0.001
	signal transduction mapk signaling pathway yeast		2.305205	AIH	< 0.001
	signal transduction phosphatidyl inositol signaling system		2.762017	AIH	< 0.001
	signaling molecules and interaction cellular antigens		3.006222	Control	< 0.001
Enzyme families	protein kinases		3.445237	AIH	< 0.001
	peptidases		4.346235	Control	0.001
Genetic information processing	folding sorting and degradation sulfurrelay system		3.433633	AIH	0.001
	restriction enzyme		3.352333	AIH	< 0.001
	folding sorting and degradation	chaperones and folding catalysts	4.222254	Control	0.001
	folding sorting and degradation RNA degradation		3.326766	Control	< 0.001
	replication and repair chromosome		4.26235	Control	< 0.001
	replication and repair DNA repair and recombination proteins		4.52453	Control	< 0.001
	replication and repair DNA replication		3.666436	Control	0.01
	replication and repair DNA replication proteins		4.232601	Control	< 0.001
	replication and repair homologous recombination		4.04477	Control	< 0.001
	replication and repair mismatch repair		3.762013	Control	< 0.001
	replication and repair nucleotide excision repair		3.663722	Control	< 0.001
	translation ribosome biogenesis		4.277014	Control	< 0.001
	translation factors		3.626632	Control	< 0.001
	protein folding and associated processing		3.633664	Control	0.001
	translation proteins		4.012673	Control	< 0.001
Glycan biosynthesis and metabolism	other glycan degradation		3.505532	AIH	0.004
	glycosyl transferases		3.65353	Control	< 0.001
	lipopolysaccharide biosynthesis		3.665456	Control	< 0.001
	lipopolysaccharide biosynthesis proteins		3.355555	Control	< 0.001
	peptidoglycan biosynthesis		4.015065	Control	< 0.001
Human diseases infectious diseases	tuberculosis		3.2522	Control	< 0.001
	Vibrio cholerae pathogenic cycle		3.006307	Control	< 0.001
Lipid metabolism	fatty acid metabolism		3.355644	AIH	0.001
	fatty acid biosynthesis		3.320655	AIH	< 0.001
	glycerol lipid metabolism		3.573666	AIH	< 0.001
	glycerol phospholipid metabolism		3.342236	AIH	< 0.001
	linoleic acid metabolism		2.663636	AIH	< 0.001
	primary bile acid biosynthesis		2.564327	AIH	< 0.001
	secondary bile acid biosynthesis		2.562635	AIH	< 0.001
	sphingolipid metabolism		3.400146	AIH	0.001
	biosynthesis of unsaturated fatty acids		3.25354	Control	< 0.001
	lipid biosynthesis proteins		3.366533	Control	< 0.001
	arachidonic acid metabolism		3.001737	Control	< 0.001
Metabolism	biosynthesis and biodegradation of secondary metabolites		2.337226	AIH	< 0.001
	Carbohydrate metabolism		3.273224	AIH	< 0.001
Metabolism of cofactors and vitamins			3.070146	AIH	0.001
Metabolism of cofactors and vitamins	pantothenate and CoA biosynthesis		3.620133	AIH	< 0.001
	porphyrin and chlorophyll metabolism		3.762301	AIH	< 0.001

	thiamine metabolism	3.32266	AIH	0.01
	ubiquinone and quinone biosynthesis	3.507762	Control	< 0.001
	folate biosynthesis	3.675646	Control	0.01
	nicotinate and nicotinamide metabolism	3.301046	Control	0.001
	one carbon pool by folate	3.723016	Control	< 0.001
	riboflavin metabolism	3.54662	Control	< 0.001
	vitamin B6 metabolism	3.405072	Control	0.004
Metabolism of other amino acids	phosphonate and phosphinate metabolism	2.365622	AIH	< 0.001
	beta Alanine metabolism	3.32754	Control	0.001
	D alanine metabolism	3.076323	Control	< 0.001
	D glutamine and D Glutamate metabolism	3.244301	Control	< 0.001
	Glutathione metabolism	3.373242	Control	< 0.001
	Seleno compound metabolism	3.565013	Control	< 0.001
Metabolism of terpenoids and polyketides	biosynthesis of siderophore group non ribosomal peptides	2.646206	AIH	0.001
	biosynthesis of ansamycins	3.017732	AIH	< 0.001
	tetracycline biosynthesis	3.22443	AIH	< 0.001
	biosynthesis of vacomycin group antibiotics	2.764016	Control	0.04
	prenyl transferases	3.634663	Control	0.001
	terpenoid backbone biosynthesis	3.652053	Control	< 0.001
	zeatin biosynthesis	2.74442	Control	0.001
Nucleotide metabolism	purine metabolism	4.423001	Control	0.001
	pyrimidine metabolism	4.3536	Control	< 0.001
Organismal systems	digestive system carbohydrate digestion and absorption	2.560013	Control	< 0.001
	digestive system mineral absorption	2.45776	Control	< 0.001
	digestive system protein digestion and absorption	2.624046	Control	0.007
Xenobiotics biodegradation and metabolism	amino benzoate degradation	3.222377	AIH	0.001
	bisphenol degradation	2.764566	AIH	< 0.001
	dioxin degradation	2.626046	AIH	< 0.001
	naphthalene degradation	3.26467	AIH	< 0.001
	xylene degradation	2.604525	AIH	< 0.001
	drug metabolism cytochrome P450	2.440127	Control	< 0.001
	nitrotoluene degradation	2.701356	Control	0.04
	drug metabolism other enzymes	3.622301	Control	0.001
	toluene degradation	3.232605	Control	< 0.001
	chloroalkane and chloroalkene degradation	3.276073	Control	< 0.001

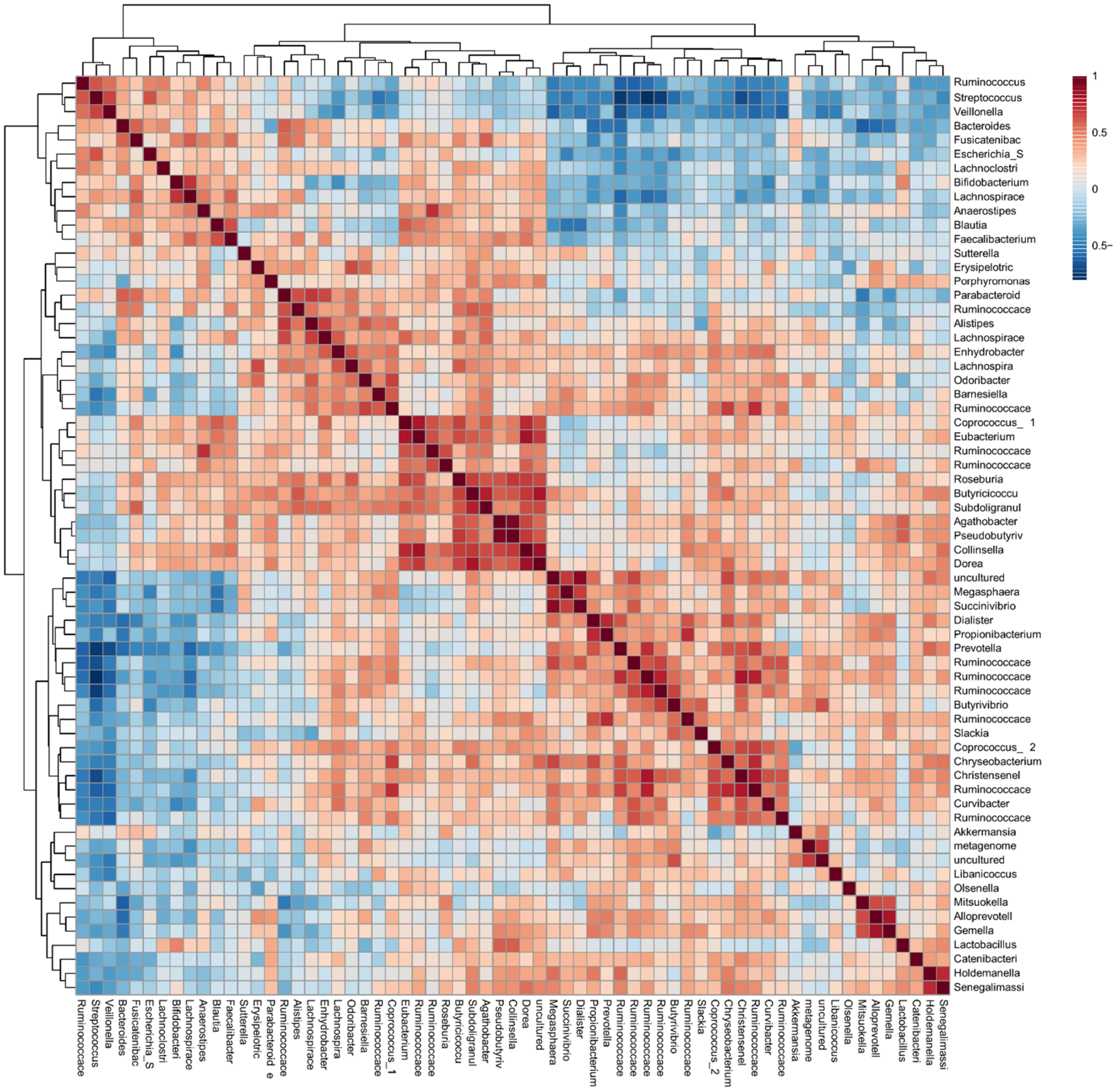

Supplemental Figure S1. Correlation plot of pairwise Spearman correlations between the top abundant genera.
